# A case report of severe myocarditis combined with erythema multiforme caused by herpes simplex virus-1

**DOI:** 10.3389/fcvm.2025.1421364

**Published:** 2025-03-12

**Authors:** Anwu Huang, Bin Lin, Xiaojun Ji, Shanjiang Chen

**Affiliations:** Department of Cardiology, Wenzhou Central Hospital, Wenzhou, Zhejiang, China

**Keywords:** herpes simplex virus, VA-ECMO, myocarditis, erythema multiforme, case report

## Abstract

Herpes simplex virus-1 (HSV-1) is a recognised pathogen that is primarily associated with skin mucosal infection. However, in rare cases, it has been observed to lead to skin extramucosal manifestations, including myocarditis. The present case report documents the occurrence of severe myocarditis in a 35-year-old female patient, attributed to HSV-1 infection. She received cardiac support in the form of VA-ECMO (Venoarterial Extracorporeal Membrane Oxygenation). Concurrently, the patient exhibited cutaneous manifestations of erythema multiforme, which demonstrated a favourable response to antiviral and hormonal therapy. This case underscores the significance of HSV-1 as a causative agent of myocarditis and underscores the necessity for vigilance and expeditious treatment of potential cardiac complications caused by HSV-1.

## 1 Introduction

Herpes simplex virus (HSV) is a common virus, divided into two types HSV-1 and HSV-2. HSV-1 usually causes infections around the mouth and HSV-2 usually causes infections around the genitals.However, herpes simplex virus can also cause life-threatening infections such as hepatitis and encephalitis (1–3).

Severe myocarditis is a serious heart disease characterized by rapid progression and serious sequelae. Severe cases can lead to heart failure, arrhythmia, and even sudden death (4). Currently common viruses associated with myocarditis include Coxsackie virus group B, adenovirus, while myocarditis caused by HSV is rarer, with an estimated prevalence of less than 1% of acute myocarditis (5).

Erythema multiforme (EM) is an immune-mediated mucous skin disease in which the rash appears as a pleomorphic, symmetrically distributed erythema with characteristic target-like lesions (6). There are many causes of erythema multiforme, including infections, cancer, medications, autoimmune diseases, and more. Of these, 90% were associated with infection (7), the most common being adult herpes simplex virus (66.7% for HSV-1 and 27.8% for HSV-2) (8).

In this case, we reported for the first time that HSV-1 infection caused both severe myocarditis and erythema multiforme, and the patient recovered after treatment and was discharged from hospital.

## 2 Case presentation

A 35-year-old female patient with no prior medical history and a normal immune system was admitted to the emergency department with fever and abdominal pain that had lasted for three days and limb convulsions that had lasted for five hours.

**Figure 1:**
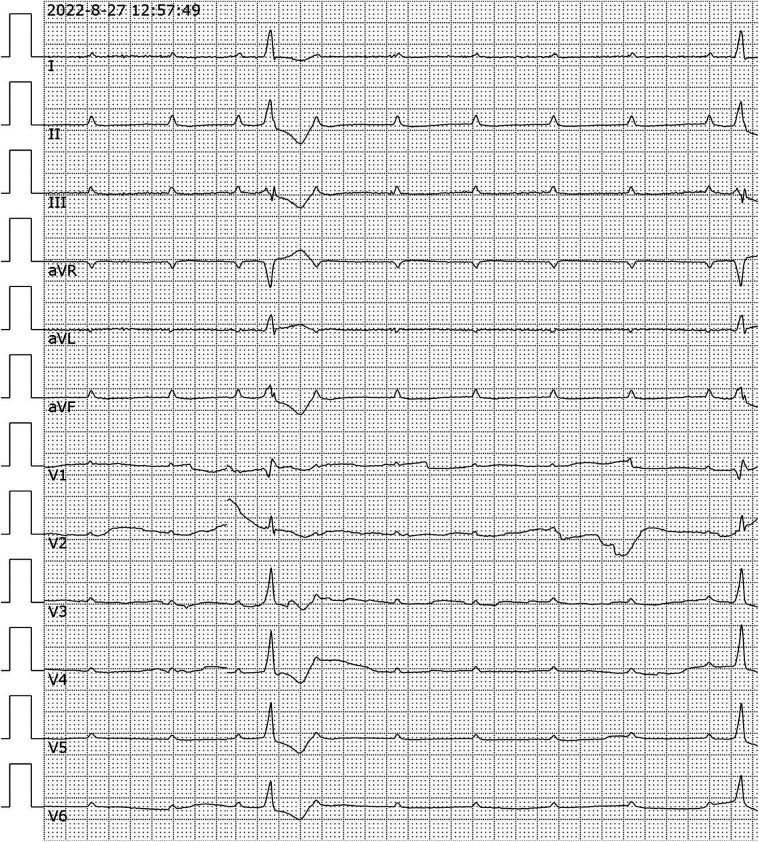
Analysis of electrocardiogram. 12-lead electrocardiogram showed high atrioventricular block. aVR, Augmented Vector Right; aVL, Augmented Vector Left; aVF, Augmented Vector Foot.

Upon admission, the patient had sudden limb twitch again, loss of consciousness, accompanied by urinary incontinence. The limb twitch lasted for about 2 min and then the patient stopped by herself. Subsequent to the patient regaining consciousness, he exhibited symptoms including fatigue and dizziness, accompanied by abdominal pain and distension. However, no obvious abnormality was found in chest and abdominal CT examination. Thus, emergency department was considered for “epilepsy” and sodium valproate was given epilepsy control. During the observation period, the patient had another episode of unconsciousness, with his eyes turned upside down, and woke up after 10 s. ECG monitoring indicates a drop in heart rate to 30 beats/min with premature ventricular beats, Blood pressure Monitoring indicates a rapid drop in blood pressure. A bedside electrocardiogram suggests a high atrioventricular block (Figure 1), Laboratory tests showed: WBC 6.8 × 10^9^/L, CRP 18.7 mg/L, ALT 181 U/L, LDH 508 U/L, cTnI 14 ng/ml, CK 629 U/L, Lactate 2.98 mmol/L, NT-proBNP 3,940 ng/L. Considering the patient's critical condition, she was admitted to the intensive care unit(ICU) for further treatment.

**Figure 2:**
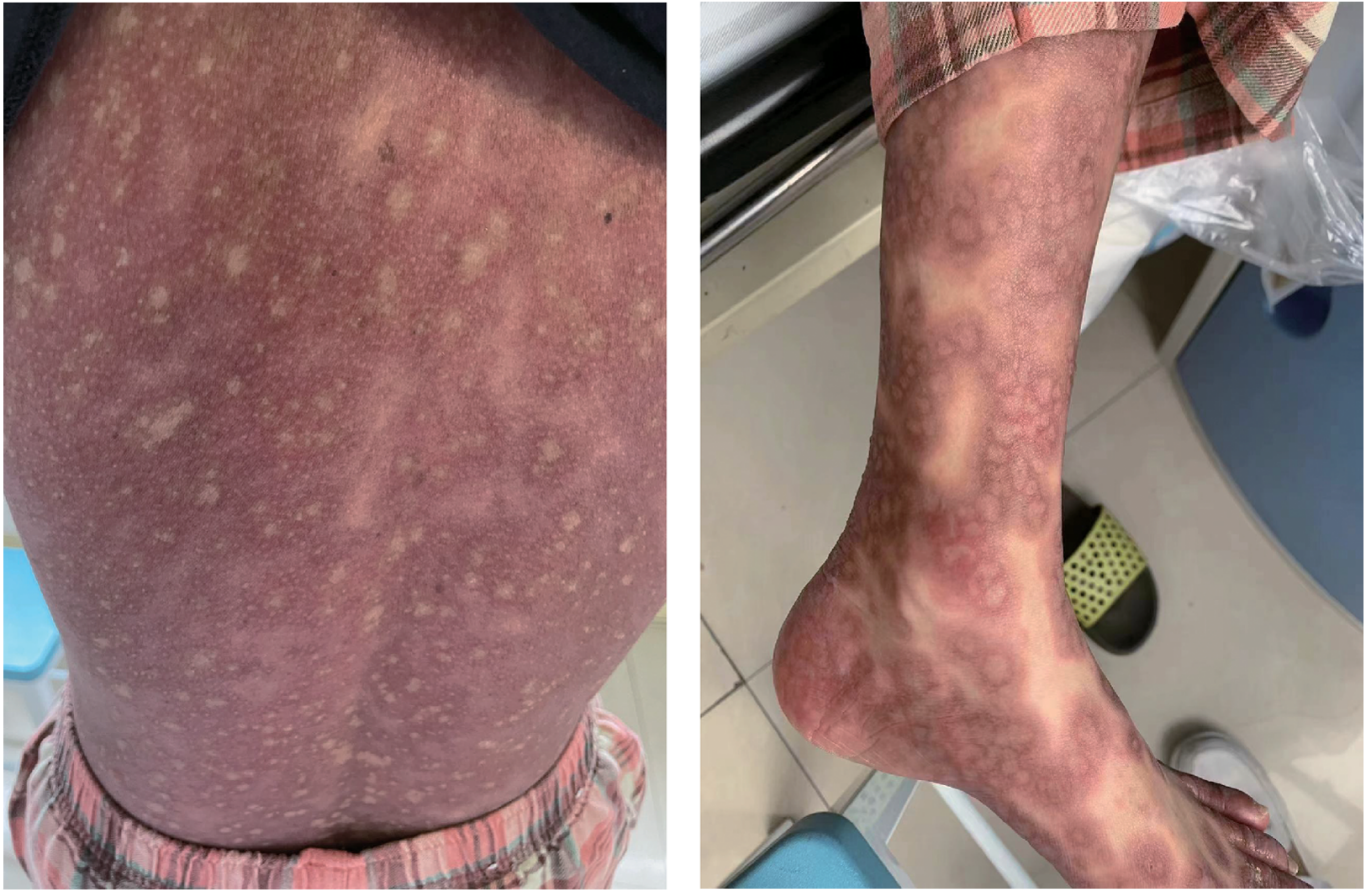
Erythema multiforme. Physical examination revealed scattered red maculae and maculopapules on the trunk and limbs.

**Figure 3:**
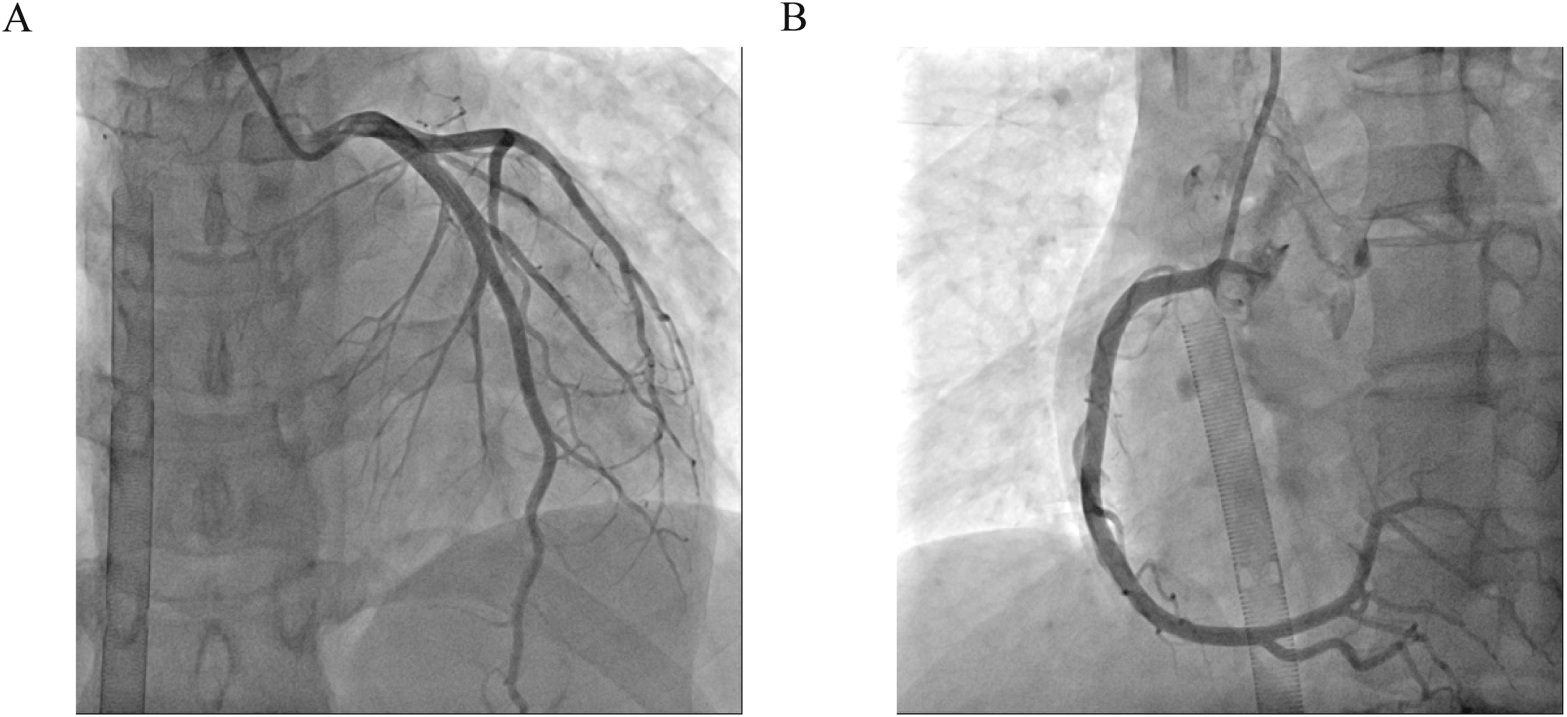
Coronary angiography. **(A)** Left coronary artery showed no obvious coronary vessel stenosis. **(B)** Right coronary artery showed no obvious coronary vessel stenosis.

**Figure 4:**
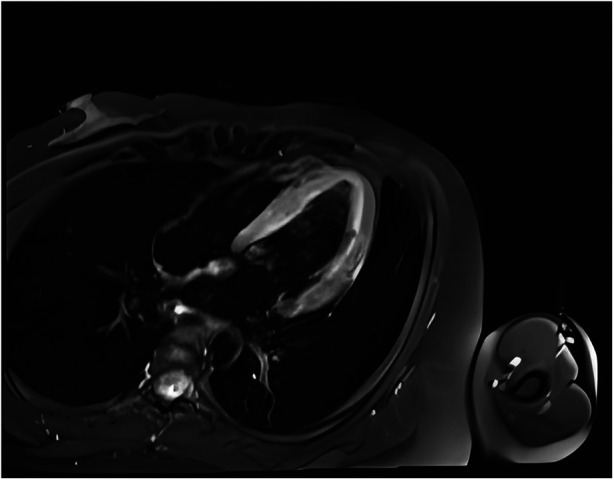
Cardiac magnetic resonance T2-weighted imaging shows diffuse hypersignal in the left ventricular wall.

One day after admission, the patient's vital signs were unstable and her blood pressure remained low despite high doses of norepinephrine and m-hydroxyamine. Physical examination revealed scattered red maculae and maculopapules on the trunk and limbs, accompanied by iridoid lesions, blisters, and oral mucosal erosion (Figure 2). Echocardiography examination indicated a significant decrease in Ejection fraction (24%). A review of laboratory tests showed: WBC 6.6 × 10^9^/L, CRP 20.2 mg/L, ALT 225 U/L, LDH 658 U/L, cTnI 20.9 ng/ml, CK 803 U/L, Lactate 6.62 mmol/L, NT-proBNP 6,690 ng/L. Considering the risk of cardiac arrest remains high, endotracheal intubation, V-A ECMO, and emergency coronary angiography were performed. Coronary angiography showed no obvious coronary vessel stenosis (Figure 3). At the same time, we performed extensive hematological tests to determine the cause of the erythema multiforme. Blood cultures were negative for bacteria and fungi. Immunological tests such as antinuclear antibodies, anti-double-stranded DNA antibodies and rheumatoid factors were negative. COVID-19 polymerase chain reaction was negative. HIV, Hep A, B, C and Treponema pallidum serologies were negative. Coxsackie virus, adenovirus, influenza A virus, influenza B virus, human parainfluenza virus, respiratory syncytial virus, Mycoplasma pneumoniae, chlamydia pneumoniae were negative. However, Chemiluminescence showed that herpes simplex virus-1 (HSV-1) was positive. Therefore, we considered that the patient had severe myocarditis and erythema multiforme due to infection with HSV-1 virus. We gave acyclovir needle antiviral and methylprednisolone needle anti-inflammatory. After the initiation of ECMO, the patient's blood pressure significantly increased and gradually stabilized at a systolic blood pressure of 90–168 mmHg and a diastolic blood pressure of 64–112 mmHg. Arterial blood gas analysis showed a significant improvement in acidosis. Therefore, we discontinued all vasoactive medications on the second day following the initiation of ECMO. Four days after the initiation of ECMO, the flow rate was reduced to 1.2 L/min, the patient's blood pressure remained relatively stable, blood gas analysis indicated good tissue perfusion, and follow-up echocardiography showed that the ejection fraction (EF) had recovered to 42%. We concluded that the criteria for ECMO withdrawal had been met, and ECMO was removed four days after its initiation. The day after ECMO was removed, the patient was then scheduled for a cardiac magnetic resonance imagery (CMRI) examination. Cardiac magnetic resonance T2-weighted imaging shows diffuse hypersignal in the left ventricular wall (Figure 4). Finally, the patient's symptoms gradually improved, the rash gradually disappeared on the seventh day, and she was discharged successfully after 2 weeks of treatment. The specific clinical process is shown in Supplementary Image 1. The patient has now been under observation for a period of one year, during which time she has attended five outpatient reviews at the hospital. No significant abnormalities have been observed in the patient's skin, troponin levels, electrocardiogram, or cardiac ultrasound. Furthermore, the patient has not continued any medications for the treatment of ring erythematosis or myocarditis.

## 3 Discussion

In this case, we report a rare case of myocarditis and erythema multiforme caused by HSV-1 infection. This patient was treated with ECMO for hemodynamic instability due to severe myocardial damage. At the same time, during the treatment of myocarditis, the patient developed erythema multiforme. Finally, under the treatment of acyclovir and methylprednisolone, the patient's vital signs gradually stabilized, the rash subsided, and the patient successfully recovered. To our knowledge, this is the first case of myocarditis combined with erythema multiforme caused by HSV infection.

Myocarditis, one of the most important diseases of cardiovascular disease, used to have an incidence of 1 to 10 cases per 100,000 people per year (9). The highest risk was found in people aged 20 to 40 (10). Myocarditis can be caused by a variety of infectious and non-infectious causes. In infectious myocarditis, viruses are the most common cause. Viral myocarditis can be caused by adenovirus, enterovirus, COVID-19 and other viruses, but HSV-1 virus caused myocarditis is extremely rare. Bowles et al. used polymerase chain reaction (PCR) to analyze viral genomes in heart tissue and blood to identify common viral causes of myocarditis in different age groups. The viral genome was amplified in 239 (38%) of 624 patients with myocarditis, of which HSV infection accounted for only 0.8% of myocarditis (5). Previous basic studies have shown that myocarditis caused by HSV-1 infection is regulated by members of the TRIM protein family. TRIM29 could regulate the innate immunity to promote DNA virus HSV-1 infection (11) and loss of TRIM29 mitigates viral myocarditis by attenuating PERK-driven ER stress immune response (12). Additionally, TRIM18 deficiency is reported to control DNA virus HSV-1 infection and viral myocarditis (13). In this case, although acute myocardial infarction caused by coronary artery occlusion was excluded by coronary angiography, and myocarditis was diagnosed by troponin, magnetic resonance, and echocardiography, it was still difficult to diagnose the cause of myocarditis. The occurrence of multitype erythema provides the basis for the diagnosis of viral myocarditis and the direction for the targeted virus screening.

Erythema multiforme is an immune-mediated disease of the skin and mucosa, usually with acute onset. The rashes are mainly distributed on the extremities and trunk, presenting as target-like characteristic skin lesions (7). The most common cause of erythema multiforme is caused by HSV virus infection, of which HSV-1 is the main cause, accounting for 66.7% (8). The most common cause of erythema multiforme is caused by HSV virus infection, of which HSV-1 is the main cause, accounting for 66.7%. At present, for acute erythema multiforme caused by infection, anti-infection treatment can be carried out, and glucocorticoids can be given to improve the symptoms of patients after the infection is controlled.

In 2024, the American College of Cardiology recommended repeated measurements of troponin and cardiac function for severe myocarditis, antiviral therapy and hormonal therapy for severe myocarditis caused by viral infection, and mechanical circulatory should be used to support for patients with hemodynamic instability (14). In this case, the patient was diagnosed with severe viral myocarditis. The treatment plan comprised acyclovir antiviral therapy and methylprednisolone anti-inflammatory therapy, in addition to ECMO for haemodynamic support, as outlined in previous guidelines. Ultimately, the patient exhibited a positive response to treatment and was discharged from the hospital, underscoring the significance of a uniform treatment approach.

Our study has several limitations that should be acknowledged. First, we did not biopsy the patient's skin lesions, and second, we did not perform an endocardial biopsy of the myocardium. As a result of these two limitations, we were unable to conduct any histopathological examinations of the patient. Additionally, although the patient had no prior history of autoimmune diseases, was not on any medications, and most all viral infections were ruled out, we still need to consider the potential impact of these three factors on our study.

## 4 Conclusions

In summary, we report a rare case of myocarditis associated with erythema multiforme due to HSV-1 infection. We are very glad that this case of severe myocarditis was able to recover and be discharged from hospital with the help of ECMO. It is important to identify the cause of severe myocarditis, and timely treatment is very necessary.

## Data Availability

The raw data supporting the conclusions of this article will be made available by the authors, without undue reservation.

## Supplementary material

Supplementary Figure S1 The timeline of clinical process.
