# Supplementary figures and images for: A case report of severe myocarditis combined with erythema multiforme caused by herpes simplex virus-1
Source: Front Cardiovasc Med. 2025 Mar 12;12:1421364. doi: 10.3389/fcvm.2025.1421364 (PMC11937124; doi:10.3389/fcvm.2025.1421364)

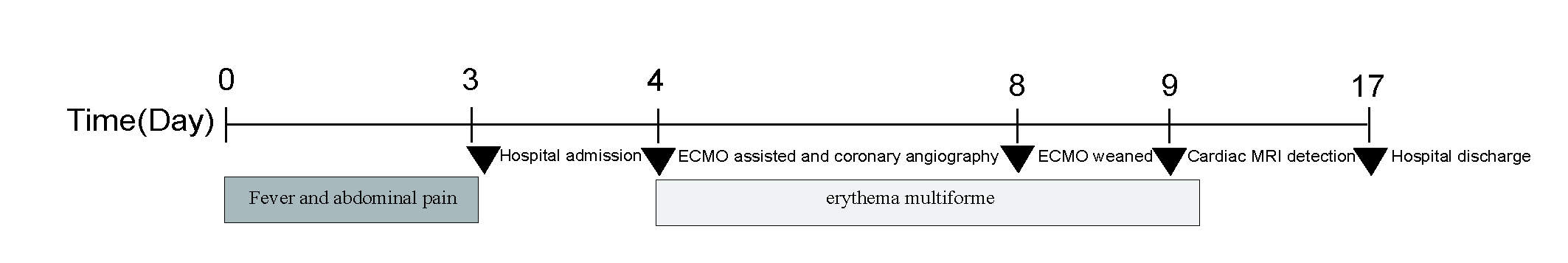

Supplement: Supplementary Figure S1 — The timeline of clinical process. [file Image1.tif]
